# Supplementary material for: The Knock-Down of the Chloroquine Resistance Transporter PfCRT Is Linked to Oligopeptide Handling in Plasmodium falciparum
Source: Microbiol Spectr. 2022 Jul 18;10(4):e01101-22. doi: 10.1128/spectrum.01101-22 (PMC9431119; doi:10.1128/spectrum.01101-22)
Supplement: Supplemental file 1 — Supplemental material. Download spectrum.01101-22-s0001.pdf, PDF file, 0.1 MB [file spectrum.01101-22-s0001.pdf]

Supplementary information

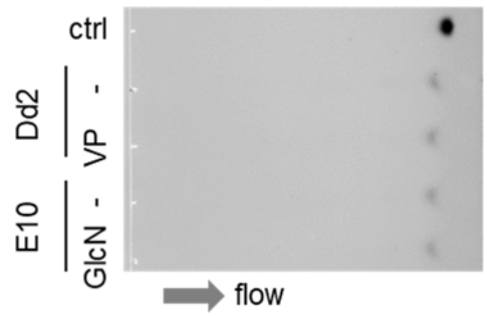

Fig S1 Thin layer chromatography showing stability of fluorescently labeled dipeptide VD during the course of a three day incubation period in cultures of E10 (in the presence and absence of glucosamine) and Dd2 (in the presence and absence of verapamil). As a control the fluorescently labeled dipeptide VD was run in parallel (ctrl).

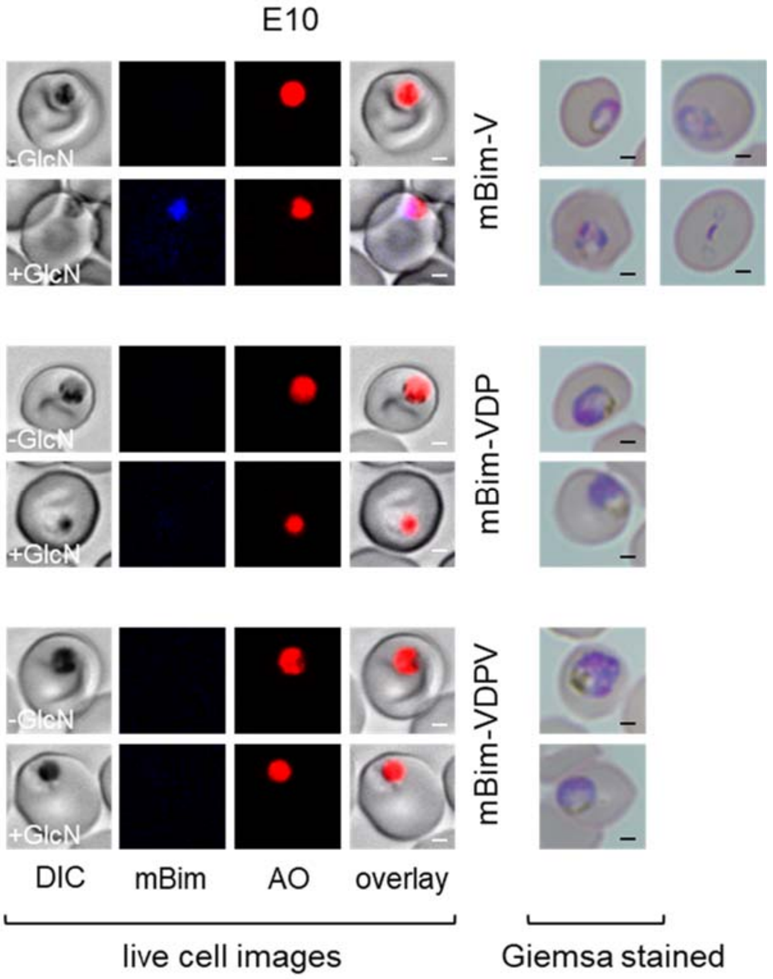

Fig S2 Accumulation of the fluorescently-labeled single amino acid valine (mBim-V) in the digestive vacuole of the conditional PfCRT knock-down mutant E10 in the presence and absence of GlcN, but not of mBim-VDP or MBim-VDPV. Representative images are shown. Acridine orange (AO) was used as live cell viability probe staining the digestive vacuole. Representative examples of Giemsa-stained parasites are shown. Bar, 1µm.

The supplementary Tables S1 to S8 are openly available at figshare at https://doi.org/10.5061/dryad.573n5tb9c

Table S1 Ideom file showing all metabolites identified in the two PfCRT knock-down mutants E10 and G7 and in the parental strain Dd2 in the presence and absence of 1 mM glucosamine for 1 day. Metabolites are listed and the file in an Excel format can be read according to instructions at (60). Sample identifier as follows: A1-A4, human red blood cells (RBC) untreated; B1-B4, RBC + GlcN; C1-C4, Dd2 untreated; D1-D4, Dd2 + GlcN; E1-E4, E10 untreated; F1-F4, E10 + GlcN; G1-G4, G7 untreated; H1-H4, G7 + GlcN. Four independent biological replicates were investigated per cell line and condition.

Table S2 Ideom file showing all metabolites identified in the two PfCRT knock-down mutants E10 and G7 and in the parental strain Dd2 in the presence and absence of 1 mM glucosamine for 3 days. Metabolites are listed and the file in an Excel format can be read according to instructions at (60). Sample identifier as follows: A1-A3, Dd2 untreated; B1-B3, Dd2 + GlcN; C1-C3, E10 untreated; D1-D3, E10 + GlcN; E1-E3, G7 untreated; F1-F3, G7 + GlcN; G1-G3, RBC untreated; H1-H3, RBC + GlcN. Three independent biological replicates were investigated per cell line and condition.

Table S3 Metabolites associated with PfCRT knock-down after 1 day of induction.

Table S4 Metabolites associated with PfCRT knock-down after 3 days of induction.

Table S5 PfCRT-associated oligopeptides related to RBC proteins and physicochemical properties. Data obtained from one day induction of the PfCRT knock down were analyzed.

Table S6 PfCRT-associated oligopeptides related to RBC proteins and physicochemical properties. Data obtained from 3 day induction of the PfCRT knock down were analyzed.

Table S7 Oligopeptides associated with PfCRT knock-down identified in a targeted approach (one day of induction).

Table S8 Oligopeptides associated with PfCRT knock-down identified in a targeted approach (three days of induction).

Table S9: Fluorescently labeled dipeptides consisting of the fluorophore monobimane (mBim) and the linker 3-mercaptopropionic acid {Mpa} that were coupled to the N-terminus (N-term) of the dipeptide. The C-terminus (C-term) remained unmodified. The structure of a typical fluorescently labeled dipeptide is shown in Fig. 6A.

N°	N-term	sequence	C-term	pool
1	MBin	{Mpa}KT	COOH	1
2	MBin	{Mpa}AQ	COOH	1
3	MBin	{Mpa}HL	COOH	1
4	MBin	{Mpa}NL	COOH	1
5	MBin	{Mpa}LS	COOH	1
6	MBin	{Mpa}GS	COOH	2
7	MBin	{Mpa}LT	COOH	2
8	MBin	{Mpa}PA	COOH	2
9	MBin	{Mpa}SP	COOH	2
10	MBin	{Mpa}VG	COOH	2
11	MBin	{Mpa}PK	COOH	5
12	MBin	{Mpa}GH	COOH	5
13	MBin	{Mpa}PV	COOH	5
14	MBin	{Mpa}LP	COOH	5
15	MBin	{Mpa}DK	COOH	5
16	MBin	{Mpa}KS	COOH	6
17	MBin	{Mpa}HK	COOH	6
18	MBin	{Mpa}MP	COOH	6
19	MBin	{Mpa}KG	COOH	6
20	MBin	{Mpa}SH	COOH	6
21	MBin	{Mpa}HL	COOH	6
22	MBin	{Mpa}QA	COOH	7
23	MBin	{Mpa}LH	COOH	7
24	MBin	{Mpa}SL	COOH	7
25	MBin	{Mpa}TL	COOH	7
26	MBin	{Mpa}GV	COOH	7
27	MBin	{Mpa}SV	COOH	7
28	MBin	{Mpa}VD	COOH	2

Table S10: Primers. Sequences used for In-Fusion cloning are highlighted in red.

primer	sequence
PfCRT-1-IF-for	G GCC CCT TTC CGC GG ATG AAA TTC GCA AGT AAA AAA AAT AAT C
PfCRT-Bgl II-rev	GA *AGA TCT* GTG TCG TTC CTA AAA AGG TC
PfCRT^{rc}-Bgl II-for	GA *AGA TCT* TTC AAC TTG CAA TTC TTC GC
PfCRT^{rc}-1280-IF-no stop-rev	AGG GTA TCC ACC GCC TTG AGT AAT AAT AGA GTC AAC G
PfCRT-3'UTR-IF-for	ACA TGT AGG AGG GGA AAT TAT ATC ATT TAT TTT TTT ATA TTT TTT
PfCRT-3'URT-IF-rev	TTA CAA AAT GCT TAA G CT TTT AAC TTT TTT TTT TTT CCC
PfCRT-guide4-IF-for	TAA GTA TAT AAT ATT CCT CTT CAG AAT TGT GGT CT GTT TTA GAG CTA GAA
PfCRT-guide4-IF-rev	TTC TAG CTC TAA AAC AG ACC ACA ATT CTG AAG AGG AAT ATT ATA TAC TTA
Primer 1	AAA TAT TTT AAA TAC GAC ATT CCG
Primer 2	TAA AAT AGT ATA CTT ACC TAT ATC
Primer 3	GAA AAT GAA GAT TCC GAA GGA GAA TTA AC
Primer 4	ACT TTG GTA ATC TGA TTT TTA ATG
Primer 5	CTC TTT AAA AAT AAG TTT AAA CAC
Primer 6	GAA AAC GAA GAC TCT GAA GGT GAA TTG
PfCRT-Pyro-for-biotin	**biotin**-GAC CTT TTT AGG AAC GAC AC
PfCRT-Pyro-rev	CAT AAT AAT AAG AAG CAG AAG AAC
PfCRT-Pyro-seq	AAC XAA AGA TTG XAT XTT XCC AGT AG
